# Exploring the association between mild behavioral impairment and plasma p‐tau217: Implications for early detection of Alzheimer's disease

**DOI:** 10.1002/dad2.70119

**Published:** 2025-05-21

**Authors:** Maryam Ghahremani, Rebeca Leon, Eric E. Smith, Zahinoor Ismail

**Affiliations:** ^1^ Department of Psychiatry Cumming School of Medicine University of Calgary Calgary Alberta Canada; ^2^ Hotchkiss Brain Institute Cumming School of Medicine University of Calgary Calgary Alberta Canada; ^3^ Department of Clinical Neurosciences Cumming School of Medicine University of Calgary Calgary Alberta Canada; ^4^ Community Health Sciences University of Calgary Calgary Alberta Canada; ^5^ O'Brien Institute for Public Health University of Calgary Calgary Alberta Canada; ^6^ Pathology and Laboratory Medicine University of Calgary Calgary Alberta Canada; ^7^ Clinical and Biomedical Sciences Faculty of Health and Life Sciences University of Exeter Exeter UK

**Keywords:** Alzheimer's disease, data‐driven cutoff, Gaussian mixture modeling, mild behavioral impairment, neuropsychiatric symptoms, plasma p‐tau217

## Abstract

**INTRODUCTION:**

Mild behavioral impairment (MBI), marked by late‐onset persistent neuropsychiatric symptoms (NPS), may signal early dementia risk. While MBI is linked to previously established amyloid‐beta (Aβ) and tau biomarkers, its association with plasma p‐tau217, a promising blood‐based biomarker for Alzheimer's disease (AD), remains unexplored. Here, we investigated the association between MBI and plasma p‐tau217 in dementia‐free individuals from the Alzheimer's Disease Neuroimaging Initiative.

**METHODS:**

MBI was defined using the Neuropsychiatric Inventory (NPI) data. Linear regression assessed the association between NPS status and continuous p‐tau217 levels, while logistic regression modeled the association between NPS status and p‐tau217 positivity, using a study‐specific cutoff. Models adjusted for age, sex, education, and cognitive diagnosis.

**RESULTS:**

Among 101 participants (mean age = 72.0 ± 6.5; 44.6% female), those with MBI had higher plasma p‐tau217 levels (*β* = 36.4%; 95% confidence interval [CI]: 2.2–82.0, *p *= 0.04) and higher odds of being p‐tau217 positive (odds ratio [OR] = 3.06, 95% CI: 1.14–8.70, *p* = 0.03) than MBI‐ participants.

**DISCUSSION:**

Findings support the role of MBI in AD risk stratification.

**Highlights:**

- Mild behavioral impairment (MBI) is linked to elevated plasma p‐tau217, a specific Alzheimer's disease biomarker.
- MBI increases the odds of plasma p‐tau217 positivity in dementia‐free individuals.
- Findings support MBI as an early indicator for Alzheimer's disease risk.
- MBI assessment can improve biomarker‐based screening and clinical trial efficiency.

## 1 BACKGROUND

Alzheimer's disease (AD) is a progressive neurodegenerative disorder that unfolds over a spectrum of clinical stages, with the accumulation of specific proteinopathies driving symptom progression. The 2024 National Institute on Aging and Alzheimer's Association (NIA‐AA) revised criteria for AD provide a biological definition based on disease‐specific core biomarkers amyloid‐beta (Aβ) and tau.^1^ While cognitive function is the core measure in the clinical evaluation of preclinical and prodromal stages of dementia, mild neurobehavioral changes may coexist and even precede cognitive decline, as highlighted in the 2018 NIA‐AA Research Framework.^2^ Mild behavioral impairment (MBI), characterized by later‐life onset of persistent neuropsychiatric symptoms (NPS) representing a change from longstanding patterns, identifies persons at risk for cognitive decline and incident dementia by incorporating natural history into risk stratification.^3^, ^4^ In addition to prognostic value for incident dementia,^5^, ^6^, ^7^, ^8^, ^9^, ^10^ MBI is associated with AD neuropathologic changes, including cerebrospinal fluid (CSF) Aβ_42_/Aβ_40_ ratio^11^ and p‐tau181,^11^, ^12^, ^13^ Aβ^14^ and tau positron emission tomography (PET)^12^, ^15^ imaging markers, and neurodegeneration.^16^, ^17^ However, both CSF and PET are costly, invasive, and not ideal for widespread clinical applications,^18^ prompting the search for more accessible diagnostic tools.

Recent advancements in AD diagnostics have led to the development of blood‐based plasma biomarkers as scalable diagnostic tools that make the biological diagnosis of AD more accessible, potentially transforming clinical research and care.^19^ Prior studies have linked MBI to plasma biomarkers such as p‐tau181,^13^, ^20^ Aβ_42_/Aβ_40_ ratio,^21^ and neurofilament light (NfL).^22^ Recently, plasma p‐tau217 has emerged as the blood‐based biomarker with the highest classification accuracy, correlating closely with Aβ and tau pathology, neurodegeneration, and cognitive impairment.^23^, ^24^ Plasma p‐tau217 has demonstrated exceptional accuracy in detecting Aβ‐PET and tau‐PET positivity,^25^, ^26^ outperforming previous plasma biomarkers, including p‐tau181, p‐tau231, Aβ_42_/Aβ_40_, glial fibrillary acidic protein (GFAP), and NfL.^24^, ^27^, ^28^, ^29^ The revised NIA‐AA criteria highlight plasma p‐tau217 as the most promising candidate to replace PET and CSF in clinical diagnostics, being sufficiently accurate to establish an AD diagnosis on its own.^1^ However, the relationship between MBI and plasma p‐tau217 remains largely unexplored. If MBI is associated with elevated plasma p‐tau217 levels, screening for MBI could serve as a simple, accessible, and noninvasive approach to help enrich samples for individuals with AD biomarker positivity. This sample enrichment could enhance clinical trial efficiency by reducing the number of trial participants needing biomarker screening, ultimately lowering costs. Furthermore, detecting MBI clinically could serve as a prompt to consider testing for plasma p‐tau217 to assess for the presence of AD pathology. The present study aimed to investigate the cross‐sectional association between MBI and plasma p‐tau217 levels and plasma p‐tau217+ status in dementia‐free older adults, advancing the understanding of how behavioral symptoms correlate with tauopathy in AD. We hypothesized that individuals with MBI would demonstrate significantly higher plasma p‐tau217 levels and higher odds of p‐tau217+ status compared to those without MBI.

## 2 METHODS

### 2.1 Study participants

**FIGURE 1:**
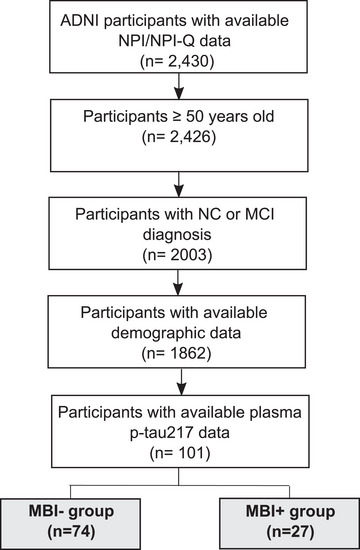
Flowchart illustrating participant inclusion/exclusion criteria. ADNI, Alzheimer's Disease Neuroimaging Initiative; MBI, mild behavioral impairment; MCI, mild cognitive impairment; NC, normal cognition; NPI, Neuropsychiatric Inventory; NPI‐Q, Neuropsychiatric Inventory Questionnaire

Participant data were from the April 2024 data release of the Alzheimer's Disease Neuroimaging Initiative (ADNI: adni.loni.usc.edu). The ADNI is a nonrandomized natural history nontreatment study launched in 2003 as a public–private partnership, led by Principal Investigator Michael W. Weiner, MD. The ADNI started recruiting participants in 2004 at 50 study sites across North America. Participants were followed up at regular intervals from baseline. Participants with mild cognitive impairment (MCI) at baseline were followed up every 6 months for the first 3 years and then yearly thereafter. Baseline normal participants were followed up every 6 months for the first year and then yearly thereafter. The ADNI has the following participant inclusion criteria: Hachinski Ischemic score ≤4; age 55–90 years; Geriatric Depression Scale score < 6; adequate visual and auditory acuity for neuropsychological testing; good general health with no diseases precluding enrollment; and minimum sixth‐grade education. Tracking the rate of conversion from normal cognition (NC) to MCI and MCI to AD is a primary outcome measure of the ADNI protocol. The ADNI Conversion Committee reviews individual participant reports and provides a consensus diagnosis. Details of clinical diagnoses have been previously described elsewhere (https://adni.loni.usc.edu/wp‐content/uploads/2010/09/ADNI_GeneralProceduresManual.pdf). Based on the ADNI clinical cognitive diagnosis, only participants who met diagnostic criteria for MCI and NC were included in the sample. All included participants had longitudinal Neuropsychiatric Inventory (NPI)^30^ or Neuropsychiatric Inventory Questionnaire (NPI‐Q)^31^ data from baseline to a follow‐up visit 6–12 months later, which was used to operationalize NPS status, as well as cross‐sectional plasma p‐tau217 measurements (version: 2023‐11‐03) (Figure 1).

### 2.2 NPS operationalization

Some participants completed the NPI, which provides both severity and frequency ratings, while others completed the NPI‐Q, which provides only severity ratings. To ensure consistency across the two instruments, the presence and severity of NPS were assessed using only the severity score, excluding frequency.^11^, ^20^, ^32^ MBI symptom severity was derived from severity scores across 10 of the 12 NPI and NPI‐Q domains using a published algorithm^5^, ^33^ adhering to the International Society to Advance Alzheimer's Research and Treatment‐Alzheimer's Association (ISTAART‐AA) diagnostic criteria for MBI. Specifically, the MBI decreased motivation domain was derived from the NPI/NPI‐Q apathy/indifference item; emotional dysregulation from the sum of depression/dysphoria, anxiety, and elation/euphoria items; impulse dyscontrol from the sum of agitation/aggression, irritability/lability, and aberrant motor behavior items; social inappropriateness from the disinhibition item; and psychosis from the sum of delusions and hallucinations items. The total MBI symptom severity score was then computed by summing the scores from the five transformed MBI domains to give a total score of 0–30, with higher scores indicating a more severe manifestation of MBI symptoms. Since NPI and NPI‐Q both assess NPS over a 4‐week reference period, the two consecutive visits MBI operational definition was applied to determine NPS status, based on whether the MBI symptom persistence criterion was met.^34^ Participants were classified as MBI+ if their total MBI symptom severity score was ≥ 1 for two consecutive study visits that were 6–12 months apart, prior to dementia diagnosis, aligning with the MBI symptom persistence criterion. Participants not meeting this criterion were classified as MBI.

**RESEARCH IN CONTEXT**

1. **Systematic review**: We reviewed prior literature on mild behavioral impairment (MBI) and its association with Alzheimer's disease (AD) biomarkers, amyloid‐beta (Aβ) and tau. While studies link MBI to established AD biomarkers, the relationship between MBI and plasma p‐tau217, a novel blood‐based biomarker with high specificity for AD pathology, was previously unexplored.
2. **Interpretation**: Our findings reveal that MBI is associated with higher plasma p‐tau217 levels and increased odds of p‐tau217 positivity in individuals with normal cognition or mild cognitive impairment. This finding strengthens the evidence that neurobehavioral symptoms are early indicators of AD proteinopathies and highlights the utility of MBI for AD risk stratification and early detection.
3. **Future directions**: Future research should explore longitudinal relationships between MBI and plasma p‐tau217 to clarify temporal dynamics and causal pathways. Additionally, using validated scales to capture MBI, such as the MBI checklist, and integrating them into biomarker‐based clinical trial designs are critical next steps.

### 2.3 Biomarker measurements

Plasma p‐tau217 was quantitatively measured in pg/ml using a novel single‐molecule array (Simoa) p‐tau217 assay developed by Janssen Research and Development.^35^ Each Simoa plate included three aliquots of two control samples obtained from participants enrolled in ADNI2/3. Participants without plasma ptau‐217 assay data prior to dementia diagnosis were excluded from the study.

### 2.4 Statistical analysis

All statistical analyses were performed using RStudio v(2024.09.0+375). Plasma p‐tau217 measurements were winsorized at 90% using the *DescTools* package to limit the influence of outliers without excluding them. Significant differences in demographic variables across NPS groups (MBI+ vs. MBI‐) were assessed based on the two‐sample *t*‐test for continuous variables and the chi‐squared test for categorical variables.

A linear regression model was fitted to examine the cross‐sectional association between NPS status as the exposure and plasma p‐tau217 level as the continuous dependent variable outcome. Since p‐tau217 concentrations were log‐transformed due to skewness, the estimated model coefficients were exponentiated to return to the original scale. To facilitate interpretation, the obtained coefficients were then reported as percentage differences in p‐tau217 levels. The model was adjusted for age, sex, years of education, and clinical cognitive diagnosis.

Additionally, logistic regression modeled the association between NPS status and plasma p‐tau217 status (positive or negative) as a dichotomized outcome. A study‐specific cutoff value for ptau‐217 positivity was determined using Gaussian mixture modeling (GMM), implemented by the *mixtools* package. GMM is a probabilistic approach that identifies normally distributed subpopulations within an overall population, with the cutoff point defined at the intersection of the fitted normal distribution clusters.^36^, ^37^ The optimal number of distributions was determined through parametric bootstrapping with 1000 repeats. Participants with plasma p‐tau217 levels higher than the data‐driven cutoff were classified as biomarker‐positive. This biomarker status was subsequently used as the outcome in the logistic regression model, while adjusting for age, sex, years of education, and clinical cognitive diagnosis.

## 3 RESULTS

### 3.1 Demographic characteristics

**FIGURE 2:**
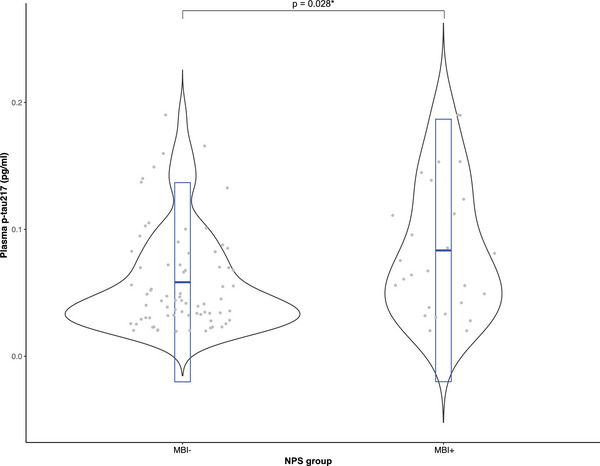
Violin plot of the distribution of plasma p‐tau217 values across NPS groups (MBI+ vs. MBI‐). Gray dots represent individual data points of p‐tau217 values, and the embedded box plot in blue represents the median, 25th, and 75th percentiles. MBI, mild behavioral impairment; NPS, neuropsychiatric symptoms

Table 1 demonstrates the sample demographic characteristics across NPS groups (MBI+ vs. MBI‐). The final sample comprised 101 participants, with 74 in the MBI‐ group and 27 in the MBI+. No significant differences were found between NPS groups in terms of age, years of education, or sex. Compared to the MBI‐ group, MCI diagnosis was more prevalent in the MBI+ group (77.8% vs. 40.5%, *p* = 0.002). Furthermore, MBI+ participants had higher levels of plasma p‐tau217 compared to MBI‐ participants (0.083 vs. 0.058 pg/ml, *p* = 0.028; Figure 2). Similarly, with dichotomized p‐tau217 measurements based on a data‐driven cutoff, the MBI+ group had a higher percentage of p‐tau217 positive participants compared to MBI‐ (66.7% vs. 39.2%, *p* = 0.026).

**TABLE 1:** Sample characteristics for MBI+ group versus MBI‐.

Parameter	Total (*N* = 101)	MBI‐ (*N* = 74)	MBI+ (*N* = 27)	*p*‐value
**Age**				
Mean (SD)	72.0 (6.5)	71.8 (6.6)	72.7 (6.3)	0.557
**Years of education**				
Mean (SD)	16.6 (2.7)	16.7 (2.6)	16.4 (2.9)	0.702
**Sex**				
Male	56 (55.4%)	39 (52.7%)	17 (63.0%)	0.489
Female	45 (44.6%)	35 (47.3%)	10 (37.0%)	
**Clinical diagnosis**				
NC	50 (49.5%)	44 (59.5%)	6 (22.2%)	**0.002**
MCI	51 (50.5%)	30 (40.5%)	21 (77.8%)	
**p‐tau217 level**				
Mean (SD)	0.065 (0.044)	0.058 (0.039)	0.083 (0.052)	**0.028**
**p‐tau217 status**				
Negative	54 (53.5%)	45 (60.8%)	9 (33.3%)	**0.026**
Positive	47 (46.5%)	29 (39.2%)	18 (66.7%)	

*Note*: p‐values were calculated based on a two‐sample t‐test for continuous variables and a chi‐squared test for categorical variables.

Abbreviations: NC, normal cognition; MBI, mild behavioral impairment; MCI, mild cognitive impairment; SD, standard deviation.

Bold *p*‐values indicate statistical significance (*p* < 0.05).

### 3.2 Cross‐sectional association of MBI and plasma p‐tau217

Linear regression demonstrated an association between NPS status and plasma p‐tau217 levels after multivariable adjustment, with MBI+ participants having 36.4% higher levels of p‐tau217 compared to MBI‐ (95% confidence interval [CI]: 2.2–82.0, *p *= 0.04) (Table 2).

**TABLE 2:** Cross‐sectional associations between MBI and plasma p‐tau217 as a continuous outcome

Outcome	Predictor	Beta^a^ (%)	95% CI	*p*‐value
Plasma p‐tau217	NPS status [MBI+]	36.38	2.22–81.96	**0.04**
	Age	2.58	0.65–4.55	**0.01**
	Sex [Male]	−10.08	−30.29–15.98	0.41
	Education	−1.94	−6.43–2.75	0.41
	Clinical diagnosis [MCI]	4.78	−19.29–36.02	0.72

*Note*: Plasma p‐tau217 values were log‐transformed. The reference groups were MBI‐ for NPS status, female for sex, and normal cognition (NC) for clinical diagnosis.

Abbreviations: CI, confidence interval; MBI, mild behavioral impairment; MCI, mild cognitive impairment; NPS, neuropsychiatric symptoms.

^a^ Beta coefficients represent the estimate percent difference in the plasma ptau‐217 biomarker level.

**FIGURE 3:**
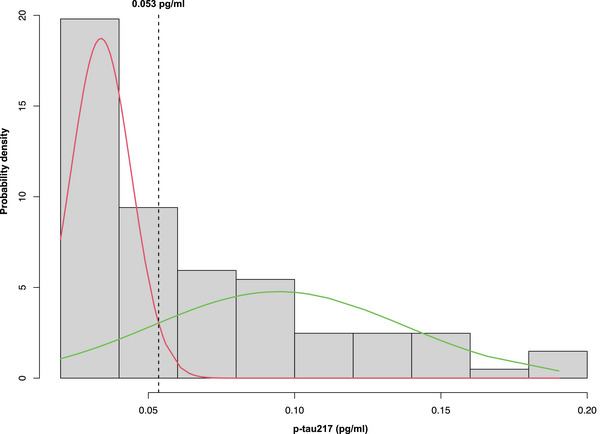
Gaussian distributions fitted using the Gaussian mixture modeling (GMM) to determine a data‐driven cutoff for plasma p‐tau217 positivity. The horizontal dashed line represents the intersection point.

Based on the GMM, the biomarker positivity cutoff for plasma p‐tau217 was determined to be 0.053 pg/mL (Figure 3). Using this cutoff, the adjusted logistic regression model demonstrated that compared to MBI‐, MBI+ participants had a significantly higher odds ratio (OR) for plasma p‐tau217 positivity (OR = 3.06, 95% CI: 1.14–8.70, *p* = 0.03) (Table 3).

**TABLE 3:** Cross‐sectional associations between MBI and plasma p‐tau217 as a categorical outcome based on a data‐driven cutoff

Outcome	Predictor	Odds ratio	95% CI	*p*‐value
Plasma p‐tau217 positivity	NPS status [MBI+]	3.06	1.14–8.70	**0.03**
	Age	1.07	1.00–1.14	0.06
	Sex [Male]	0.81	0.33–1.94	0.64
	Education	0.92	0.78–1.08	0.31
	Clinical diagnosis [MCI]	1.07	0.44–2.63	0.87

*Note*: The reference groups were MBI‐ for NPS status, female for sex, and normal cognition (NC) for clinical diagnosis.

Abbreviations: CI, confidence interval; MBI, mild behavioral impairment; MCI, mild cognitive impairment; NPS, neuropsychiatric symptoms.

## 4 DISCUSSION

The present study explored the association between MBI and plasma p‐tau217 levels in older adults with NC or MCI. Our findings revealed that individuals with MBI exhibited significantly higher plasma p‐tau217 levels and higher odds of being biomarker positive, compared to their MBI‐ counterparts. These results provide further evidence that MBI is associated with early AD‐related pathological changes, adding to the evidence base for appropriately measured behavioral symptoms representing AD proteinopathies, and underscoring the potential role of MBI as an early marker of disease.

Our findings build upon previous research linking MBI to both Aβ and tau pathologies. Notably, studies have demonstrated that MBI in dementia‐free older adults is associated with elevated levels of CSF p‐tau181, total tau, and lower levels of Aβ_42_ and Aβ_42_/Aβ_40_ ratio.^11^, ^12^, ^13^ Furthermore, imaging studies in dementia‐free older adults have demonstrated that greater MBI severity is associated with higher uptake of Aβ‐PET^14^, ^38^ and tau‐PET^12^, ^15^ tracers in cortical regions implicated in early stages of AD. Longitudinally, higher MBI total scores at baseline predicted higher Aβ deposition in the brain and faster elevation of Aβ burden over time.^38^ These findings emphasize the relationship between MBI and core AD pathologies, supporting the notion that appropriately ascertained NPS may emerge as early manifestations of underlying disease processes. Importantly, studies that included non‐MBI NPS groups (i.e., NPS not meeting MBI criteria because they were transient and not persisting across more than one study visit) found that this group either did not differ from the no NPS group or exhibited an effect that was intermediate between no NPS and MBI groups.^11^, ^20^ This finding suggests that MBI refines the approach to assessment of NPS, to capture symptoms that are more likely to reflect the behavioral manifestations of a neurodegenerative disease. In contrast, conventional approaches capture transient or reactive NPS triggered by life events or medical conditions unrelated to dementia, as well as symptoms attributable to psychiatric conditions.^39^

Plasma biomarkers offer a relatively noninvasive and cost‐effective alternative to traditional CSF or imaging biomarkers, with recent studies highlighting the utility of plasma p‐tau217 as the most accurate blood‐based biomarker for detecting AD. In a study across three independent observational cohorts, plasma p‐tau217 demonstrated high accuracy in predicting abnormal Aβ‐PET and tau‐PET signals, outperforming all previous plasma biomarkers, notably p‐tau181, p‐tau231, Aβ_42_/Aβ_40_, GFAP, and NfL and their optimal combinations.^24^ Longitudinally, annual increases in plasma p‐tau217 levels were observed exclusively in individuals positive for Aβ, with the greatest increase seen among those who also tested positive for tau.^24^ Similar findings were reported in other studies of preclinical AD samples.^26^, ^40^, ^41^ These studies indicate that plasma p‐tau217 alone may be sufficient to diagnose AD, as suggested by the 2024 NIA‐AA revised criteria,^1^ offering a relatively noninvasive and cost‐effective diagnostic tool for early detection. Indeed, TRAILBLAZER‐ALZ‐3, a study of donanemab in preclinical AD, utilized only biomarker positivity on the Janssen plasma p‐tau217 assay—the same assay data used in the present study—for study inclusion.^42^

The present study is the first to report an association between plasma p‐tau217 and MBI in a dementia‐free population. Past literature has provided evidence on the associations of MBI with previously established plasma biomarkers of AD. Both cross‐sectionally and longitudinally, MBI in dementia‐free older adults was associated with higher plasma p‐tau181 levels.^20^ Similar findings were reported in a more recent study of older adults with subjective cognitive decline (SCD) or MCI, with a steeper increase in plasma p‐tau181 in individuals with MBI psychosis domain symptoms.^13^ Furthermore, higher MBI total scores in dementia‐free older adults have been associated with lower plasma Aβ_42_/Aβ_40_ ratio, with domain‐specific associations reported for MBI‐affective dysregulation.^21^ Another longitudinal study reported a greater increase in plasma NfL levels over 2 years in individuals with MBI compared to those without.^22^ Our findings on the association of MBI with plasma p‐tau217 add to this growing evidence base, reinforcing the idea that NPS meeting MBI criteria reflect underlying pathology early in the disease course.

Both linear and logistic regression models in the present study demonstrated higher plasma p‐tau217 levels and odds of p‐tau217 positivity in MBI+ participants compared to MBI‐. Using GMM, we identified a data‐driven threshold of 0.053 pg/mL for Janssen plasma p‐tau217 positivity. This approach offered a refined, population‐specific cutoff that enhanced the accuracy of biomarker categorization for detecting AD‐related tauopathy. The previously reported plasma p‐tau217 cutoffs using the Janssen assay are in a similar order of magnitude. A two‐cohort study of dementia‐free older adults identified Janssen plasma p‐tau217 cutoffs of 0.07 pg/mL for Aβ+ status and 0.08 pg/mL for predicting progression to AD dementia.^43^ Another study across the cognitive continuum derived a threshold of 0.083 pg/mL for Janssen plasma p‐tau217 to differentiate Aβ‐PET positive from negative individuals.^44^ Notably, a study that reported a threshold identical to ours examined sex‐specific differences in the prognostic value of Janssen plasma p‐tau217 for cognitive decline and brain atrophy. In this study, levels above the threshold of 0.053 pg/mL predicted declines in verbal memory and medial temporal lobe volume in cognitively unimpaired females. Secondary analyses of Aβ‐PET data from this study further determined that this threshold corresponded to early cortical Aβ aggregation.^45^ These findings align with the utility of MBI for early detection of AD, as supported by our study. Further, these data highlight the importance of using data‐driven approaches, such as GMM, to capture early subtle changes in biomarker levels that broader, previously established cutoffs may overlook. Tailoring cutoffs to the specific population improves accuracy and ensures greater relevance for clinical decision‐making and early disease intervention.

Across both linear and logistic regression models in our study, MCI status was not significantly associated with plasma p‐tau217 levels or positivity, which may seem unexpected. However, this finding is likely influenced by the composition of the ADNI cohort, which is enriched for amyloid‐positive individuals. As a result, even participants classified as cognitively normal are likely to be on the AD trajectory, reducing the contrast between NC and MCI groups. Given that p‐tau217 is considered an early biomarker that emerges alongside amyloid changes in the preclinical stage of AD,^40^, ^41^ its levels may already be elevated in cognitively normal participants, making it more challenging to detect significant differences between NC and MCI. In a more population‐representative sample, where the NC group includes fewer preclinical AD cases and more amyloid‐negative individuals, we might expect a clearer distinction in p‐tau217 levels between NC and MCI. Furthermore, the limited number of ADNI participants with p‐tau217 testing constrains the generalizability of our findings and limits the ability to conduct interaction analyses for stratification across cognitive groups. Future studies with larger samples will be better positioned to replicate and clarify these associations, particularly with respect to MCI status.

From a clinical trial perspective, enriching samples with biomarker‐positive individuals is essential for trial efficiency and cost‐effectiveness. By demonstrating an association between MBI and elevated plasma p‐tau217 levels in a dementia‐free sample, our study highlights the potential of using MBI as a clinical tool to pre‐select individuals who are more likely to exhibit AD pathology. The utility of MBI to identify biomarker‐positive individuals has the potential to make clinical trials more accessible and cost‐effective by implementing simple, inexpensive, and even remotely administered clinical screens as a way to identify a high‐risk group suitable for biomarker testing.

This study was not without limitations. First, the cross‐sectional design limited our ability to infer causality or the temporal relationship between MBI and plasma p‐tau217 levels. The small number of ADNI participants who have undergone p‐tau217 testing is another notable limitation of the present study. As the size of this group increases in ADNI in the future, replication of this study in larger samples will be warranted to confirm and expand upon our findings. The use of the NPI/NPI‐Q with a 4‐week reference range to operationalize MBI was another notable constraint. While the two consecutive visits approach^34^ was applied to operationalize the MBI symptom persistence criterion, it did not necessarily capture behavioral changes that persisted beyond the 4‐week reference range of NPI/NPI‐Q. Another limitation was the inability to conduct domain‐level analysis for MBI, which was hampered by both the small sample and the inherent complexities of operationalizing MBI symptom persistence across each domain using the NPI/NPI‐Q. Future studies could address these issues by utilizing the MBI checklist (MBI‐C),^46^ a validated scale specifically designed to capture MBI criteria, accounting for symptom natural history. This approach would allow for more comprehensive and accurate assessments of late‐life persistent NPS across all domains of MBI.

## 5 CONCLUSION

Our findings suggest that MBI is associated with elevated plasma p‐tau217 levels in dementia‐free older adults, supporting the notion that MBI could serve as an early non‐invasive marker of AD pathology. This association has important implications for the early detection of AD and the design of more efficient clinical trials by utilizing MBI assessment as an accessible tool to identify a high‐risk group suitable for biomarker screening.

## CONFLICT OF INTEREST STATEMENT

Z.I. has served as an advisor/consultant to CADTH, Eisai, Lilly, Lundbeck/Otsuka, Novo Nordisk, and Roche. E.S. has provided consulting (unpaid) to Alnylam, Eisai, and Lilly. M.G. and R.L. report no conflicts of interest relevant to this manuscript. Author disclosures are available in the Supporting Information.

## CONSENT STATEMENT

All ADNI participants provided informed consent to participate in the study, and the ethics committee approval to conduct this study was received at contributing ADNI sites.

## Supporting information



Supporting Information
